# A possible extension to the RInChI as a means of providing machine readable process data

**DOI:** 10.1186/s13321-017-0210-6

**Published:** 2017-04-11

**Authors:** Philipp-Maximilian Jacob, Tian Lan, Jonathan M. Goodman, Alexei A. Lapkin

**Affiliations:** 1 Department of Chemical Engineering and Biotechnology, University of Cambridge, Philippa Fawcett Drive, Cambridge, CB3 0AS UK; 2 Department of Chemistry, University of Cambridge, Cambridge, CB2 1EW UK

## Abstract

The algorithmic, large-scale use and analysis of reaction databases such as Reaxys is currently hindered by the absence of widely adopted standards for publishing reaction data in machine readable formats. Crucial data such as yields of all products or stoichiometry are frequently not explicitly stated in the published papers and, hence, not reported in the database entry for those reactions, limiting their usefulness for algorithmic analysis. This paper presents a possible extension to the IUPAC RInChI standard via an auxiliary layer, termed *ProcAuxInfo*, which is a standardised, extensible form in which to report certain key reaction parameters such as declaration of all products and reactants as well as auxiliaries known in the reaction, reaction stoichiometry, amounts of substances used, conversion, yield and operating conditions. The standard is demonstrated via creation of the RInChI including the *ProcAuxInfo* layer based on three published reactions and demonstrates accurate data recoverability via reverse translation of the created strings. Implementation of this or another method of reporting process data by the publishing community would ensure that databases, such as Reaxys, would be able to abstract crucial data for big data analysis of their contents.

## Background

In the current environment of ever increasing amounts of available chemical data both industrial and academic actors find themselves in a constant process of having to review the continuously changing state-of-the-art of their activities. In 2005 it was estimated that 1.5 million new compounds alone were being discovered annually [1]. Though this figure is slightly out-of-date, it gives an estimate of the growth rate observed and the challenges this raises when trying to keep an overview of a field of research or of practice. This trend towards higher availability of data has also seen the advent of large scale databases holding chemical reaction information, such as Reaxys (Elsevier), the CAS databases accessed through SciFinder (American Chemical Society) or ChemSpider (Royal Society of Chemistry). Data held in well-structured databases are amenable to algorithmic analyses. It has been postulated in 1990 [2] and demonstrated in 2005 [3] that data held within Reaxys (or rather its predecessors) can be converted into a network, allowing the use of graph theoretical approaches. Having a network of reactions rather than a database greatly facilitates the identification of possible synthetic pathways by using network traversal algorithms [4]. Similarly, it has been shown that the network representation can be used for the optimisation of parallel syntheses [5], the identification of suspicious purchases of precursors to controlled substances [6], the estimation of functional group cross-influence on chemical reactivity [7], or the discovery of one-pot reactions [8]. These demonstrated uses rely on connectivity data across disjoint papers and some structural information on the molecules.

Particularly from a chemical engineering or process chemistry perspective, however, it is crucial to ensure that the connectivity exploited for synthesis route planning is not superficial but that the algorithms navigate the network in a meaningful way. This definition of “meaningfulness” can necessarily be adapted to the specific use case, though could encompass criteria such as economic factors, preservation of certain chemical structure elements across the route, minimisation of process condition changes between synthesis steps, or the consideration of different sustainability criteria. We have recently demonstrated the use of sustainability criteria in this context by linking a process synthesis on the basis of network traversal, with exergy analysis, automated e-factor calculation and multi criteria decision making [9]. However, such detailed analysis of reactions requires reaction data and information on the process conditions. When analysing a set of 33.5 million reactions downloaded from Reaxys [10], which amounts to 80% of the total number of reactions contained in the database [11], and removing all incomplete and multistep reactions, which leaves 15.4 million reactions or 37%, it is discovered that a significant number of data points is missing, making any further analysis impossible. We expect that any other large scale database of chemical data would, at present, have similar data scarcity issues.

**Fig. 1:**
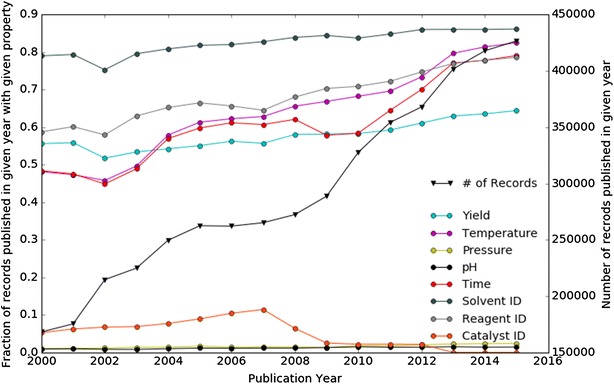
Plot of the number of records added to Reaxys in a given year and their information content when analysing a fixed set of properties. The “# of Records” line is plotted against the right-hand side y-axis

As Table 1 clearly shows, in the analysed sample set 54% of reaction entries had no yield data attached, while 53.9 and 98.4% had no temperature or pressure entries, respectively. Furthermore, the database does not record stoichiometry. The absence of such crucial data makes any automated evaluation of a synthesis route candidate along mass- or energy/exergy-based criteria nearly impossible. Analysing the multi-year trend by investigating the information content of all reactions added to Reaxys in a given year for the set of reaction data types shown in Table 1, it becomes apparent that the picture overall is encouraging in many areas, see Fig. 1. The number of records added every single year has more than doubled between the years of 2000 and 2015. During this time the information content of most entries seems to be rising for the properties analysed here. While in the year 2000 50% of records added were still without temperature data, to pick but one property, by 2015 this has dropped to roughly 20%. This trend is pointing in the right direction but 20% is still a large number and progress for many other properties, such as yield, which still hovers around 40%, has not been as good. Though awareness of and efforts to overcome the problem seem to have led to improvements, a systemic issue still seems to persist.

**Table 1:** Analysis of reaction data content in Reaxys, based on a sample set of 15.4 million reactions

Property	Percentage of reactions with value for property
Yield	46.0
Temperature	46.1
Pressure	1.6
pH-value	1.0
Reaction time	48.4
Solvent ID	70.9
Reagent ID	67.8
Catalyst ID	4.3

The cause for this problem is two-fold. On the one hand crucial data, such as reaction stoichiometry, is too frequently absent from publications, while on the other hand existing data, such as temperature or pressure which will be reported in some form in almost all papers, is not published in a way that allows it to be excerpted correctly. Both causes can be remedied. For example, by agreeing on clear and enforced data reporting standards the life of authors would be made easier by clearly setting out what data, and in which format, are required to allow the publication to achieve its maximum impact. At the same time the task of the database provider would be simplified by ensuring that the agreed, and provided, data are available in a machine-readable format.

Structure and reaction data formats can be roughly split into two categories, both of which are based on connection tables: those that are XML-based, such as the Chemical Markup Language [12, 13] and Reaxys’s internal data storage format, and those that are line-based, such as SMILES (simplified molecular input line system) [14] and InChI [15].

Connection tables are widely used and form the basis of many other standards, but no formal standard exists for the tables [16]. Connection tables store information on the atoms, bonds and, optionally, the atoms’ coordinates for a given molecule, making it a graph representation of a molecule [17, 18]. Connection tables can be canonicalised to provide one unique table per molecule, for example, using the Morgan algorithm, first proposed in 1965 and still in use with some modifications [19, 20]. By applying graph theoretical algorithms it is then possible to carry out substructure matching across a database of connection tables [16]. One of the earliest mentions of connection tables was in 1957 [21]. Subsequently, the tables found wide adoption and are used by the CAS database as well as other data formats, such as the Chemical Mark-up Language (CML) and as a basis to generate InChIs [17, 22–24]. A consequence of the way bonds are represented in traditional connection tables is that they struggle to represent delocalised bonds, inorganics and reaction intermediates, which is something that has seen some attempts at being addressed [16, 25]. In the absence of a non-proprietary standard gaining traction over time, the CTfile [26] has become the de facto standard for connection tables and the exchange of structural data [16]. It was initially developed by MDL which is now owned by Biovia, a subsidiary of the Dassault Group. This connection table forms the basis for many formats, such as of the molfile, which describes a single molecule, the reaction file (rxnfile), which contains the structural information of the reactants and products, and the Reaction-data files (RDfiles), which can represent molecules and reactions as well as their associated data. The current version of the standards can be found on Biovia’s website.

XML-based data standards are useful when it comes to electronic database storage of data as they are highly extensible, flexible and all data entries are labelled. A key example of this is the Chemical Markup Language or Elsevier’s Unified Data Model. This is useful when it comes to exchanging data between different software suites [27–29]. A key downside is that, if the data is not already generated by a machine, generation of a valid XML document can be complicated and requires a certain degree of IT knowledge.

SMILES is one of the major formats seeking to condense this tabular format into a more compact and easier to use linear, alphanumeric string [18]. This greatly reduces the required storage space and is faster than handling a whole connection table [16]. Conversion to line notation from connection tables does, however, incur some information loss [16].

An issue that very quickly arose, however, was that SMILES strings in use were not canonical, which severely limited the applicability of SMILES in databases [23]. Canonical SMILES strings are available but are proprietary and the algorithm is not publicly available. Thus, various different versions are in circulation and implementation is seriously hampered [15, 16, 30]. These severe drawbacks were among the factors that led to the creation of the IUPAC International Chemical Identifier (InChI) in order to create a freely available, non-proprietary identifier to allow the easier linking of data compilations and the unambiguous identification of chemical substances [31].

The InChI is a representation that allows for the canonical encoding of structures, with both known and, as of yet, unknown [32], tautomers and isotopes. In addition, it is an open standard and can be easily incorporated into in-house software [1]. The InChI has turned into a widely adopted, worldwide standard as far as line notation is concerned [15, 22]. Additionally, it can be hashed to further reduce required storage space and to facilitate indexing and searching [15, 16, 18, 33]. Though collisions of keys are possible due to the hashing, so far only two cases have been reported since 2007 [22]. In theory the probability is finite, but extremely small [22]. The collision resistance was investigated experimentally, with a conclusion “the current design and implementation seem to meet their goals” [34].

The InChI algorithm itself can, to date, process organometallic and coordination compounds as well as radicals, neutral and ionic organic molecules. Projects are being undertaken to extend the representation to reactions and polymers, which is facilitated by the fact that due to its hierarchical nature new layers can be added relatively easily [16, 35].

The InChI is composed of six hierarchical layers, where each successive layer is designed to provide further structural refinement [16, 32, 36]. All layers aside from the main one are optional, and will only appear if the corresponding information has been provided in the source file [16, 36]. If the same structure has been drawn at two different levels of detail, the InChI for the one with less detail forms a subset of the one with more [15]. For further technical information on InChIs the reader is referred to [37].

Amongst several extensions to the InChI agreed upon by the InChI Trust [38] is a reaction identifier termed RInChI. Largely developed by Jonathan Goodman, Chad Allen and Guenter Grethe this culminated in the publication of an interim report in 2013 [35]. The RInChI consists of a version field (*V*), three groups containing molecules (*group1* and *group2*, each containing the molecules on one side of the arrow in the reaction equation and *group3* containing the substances present above, below or on both sides of the arrow, such as solvents and catalysts) and an optional directionality layer showing whether *group1* contains the reactants and *group2* the products (denoted by “d+”), vice versa (“d−”), or if it is an equilibrium reaction (“d=”). The molecules within each group are represented by their InChIs, separated by a double forward slash “//” and are sorted; subsequently, the order of the groups containing the starting materials and products is determined using the Unix ‘sort’ command [35]. For the exact definition of version 0.02 the reader is referred to [35]. A new version (0.03) has recently been released, the definition of which can be found in [39]. A template is shown in Eq. (1):

1 $$RInChI = 0.03.1S/group1 < > group2 < > group3/directionality$$

The “0.03” denotes the RInChI version and “1S” the InChI version used. The RInChI standard, under its current scope, does not define fields to store reaction conditions, scale, process type and kinetic data, all critical for any process calculations. The RInChI has the great advantage that it is an entirely open-source standard, building on the widely-adopted InChI and supported by both IUPAC and several major publishing houses. This presents tangible advantages to the proprietary data standards in its ease of adoption and incorporation into in-house software suites. It is understood that XML-based standards are able to capture a greater wealth of data and are better suited to use in databases. This, however, comes at a cost. Firstly, permitting a near-unlimited choice of data to include and an ability to specify units relatively freely results in a lesser engagement of the publishing author with his or her data during publication. Secondly, adoption of an XML-based format is more complicated and requires a greater degree of IT proficiency. The latter point weighs heavily as it has the potential to significantly hinder uptake of a proposed standard. Using the already in-built facility to extend RInChi through auxiliary layers we put forward a potential formal interface between authors, publishers and database providers, ultimately also contributing to the quality of data stored in XML-based datasets.

In this paper we show how an optional auxiliary field appended to the RInChI, termed *ProcAuxInfo*, could be used for this purpose and demonstrate data integrity upon reverse translation in three examples, before proceeding to show a plausible application of machine readable process data in automated reaction analysis by using the reverse translated data to determine a reaction mass efficiency. To our knowledge this is the first publication trying to provide this additional information in the RInChI standard, and is intended to contribute to the discussion of standards for publication of research data in machine readable formats.

### ProcAuxInfo

#### Definition of a standard

So as to not affect the integrity of the RInChI standard it is proposed that the reaction information is appended to the existing RInChI string and that this field is optional as far as the standard is concerned. In order for the standard to be useful in addressing the challenges set out above, it requires widespread adoption, most easily achieved by demonstrating its use in extending the reach of a paper and by journals mandating submission of the data required to compile it during the editing processes.

The *ProcAuxInfo* string is to contain some of the reaction data deemed most essential to further analysis, though is open to further extension during subsequent iterations:

- Version of *ProcAuxInfo*
- Starting material
- Stoichiometry
- Reaction temperature
- Reaction pressure
- (Time: Conversion) pairs
- Yield of product and byproducts
- Molar amounts of reactants used
- Amounts of *group3* compounds used
- Reactor volume

The *ProcAuxInfo* field begins with a double dollar sign (“$$”) to clearly demarcate it from the main RInChI, as neither Version 0.03 nor Version 0.02 of the RInChI contain any dollar signs in the standard, and additional *ProcAuxInfo* layers, as outlined further on. Each field is to be separated by a single vertical line (“|”), thus taking the following form, Eq. (2):

2 $$\begin{aligned} & ProcAuxInfo \, = \, \$ \$ Version \, | \, Starting \, Material \, | \, Stoichiometry \, of \, group1| \\ & Stoichiometry \, of \, group2 \, |Temperature \, | \, Pressure \, | \\ & Time \, :Conversion \, |Yield \, | \, Amount \, of \, group1 \, \it{fed} \, | \\ & Amount \, of \, group2 \, fed \, | \, Amount \, of \, group3 \, \it{fed} \, | \\ & Volume \, of \, reactor \\ \end{aligned}$$

If no data are available for a given field, or sub-field, a question mark (“?”) is to be used as a space-holder instead. If a given group is absent from the RInChI, for example if no auxiliaries are used and thus no group3 exists, then the fields in the ProcAuxInfo relating to the missing group are to contain a question mark too. The current version is 0.01. The version field is to have exactly one decimal point at all times and is to begin with “PAI” to clearly identify the following block.

The “starting material” is the species with respect to which all properties, such as conversion and yield, are specified. This may be the limiting reactant but does not have to be. It is to be specified by its group number followed by the index of its position in that group counting left to right, separated by a colon (“:”). It is realised that since different studies of the same reaction may define different substances as starting materials, the ProcAuxInfo layer will not be canonical. Since reaction searching is, however, carried out through the canonical InChI string and the ProcAuxInfo layer acts as data repository this is not considered to create any problems.

The stoichiometry fields are based on the stoichiometric coefficients of the products and reactants as found in the fully balanced stoichiometric equation, based on which the RInChI is compiled. These are to be integers and positive, as the directionality is already given in the main body of the RInChI. The coefficients are to be listed according to the order of the corresponding species in the respective *group* and separated from each other by use of a semicolon (“;”).

Reaction temperature is to be given in degrees Kelvin and the reaction pressure in Pascals. The reaction pressure is to be represented in scientific exponential notation in order to save space and to clearly indicate the number of significant digits.

Time is to be specified in seconds, again in scientific exponential notation. Reaction time is often reported as time taken to achieve maximum conversion, though different definitions are possible and the definition used in the particular case is thus not always apparent. For this reason, time is reported as a value pair along with conversion of the starting material. The two values are to be separated by a colon (“:”). To allow kinetic studies it is encouraged to publish multiple time:conversion pairs, each separated by a semicolon (“;”). In the case of a flow experiment residence time:conversion pairs are to be published instead. Both yield and conversion values are to be published in their decimal fractional value out of one rather than as percentage (for example, 0.01 instead of 1%). The yield is to be included for each species derived from the starting material. The yields are to be listed in the order in which the respective products are listed in *group1* or *group2* and separated by a semicolon (“;”). Where a substance is not derived from the starting material and a yield would thus be meaningless or where no yield data are available, the field for that substance is to contain a question mark (“?”) as a space holder instead. The yield is to be calculated using the following equation:

3 $$Y_{i} = \frac{{n_{i,out} - n_{i,in} }}{{n_{SM,in} }}$$

where *Y*_*i*_ is the yield of species *i*, *n*_*i*,*out*_ is the amount of *i* at the end of the reaction and $$n_{i,in}$$ is the amount fed (in the case of the flow reactions these are the corresponding flow rates); *n*_*SM*,*in*_ is the amount of starting material fed. The conversion is defined as:

4 $$X = \frac{{n_{SM,out} - n_{SM,in} }}{{n_{SM,in} }}$$

Amounts of *group1*, *group2* compounds are to be specified in terms of moles of substance fed (or mol s^−1^ fed in the case of flow reactions) and listed in the order that the compounds are given in the respective *group* in the main body of the RInChI. The different values are to be separated using a semicolon (“;”) and given in scientific exponential notation.

For catalysts it may not be meaningful to specify the amounts in moles as it is not always clear what constitutes a molecule of the catalyst. Thus, the catalyst is specified in grams as a base unit. In addition, in the case of flow chemistry or bulk continuous processes the catalyst might be immobilised and thus does not have an associated flowrate, for example in fluidised catalytic beds, coated wall reactors or packed beds. As such, each entry in *group3* is to be followed by “:$$\texttt{m}$$” or “:$$\texttt{g}$$”, depending on whether or not it is expressed as moles or grams and subsequently by “:$$\texttt{f}$$” or “$$\texttt{:a}$$” depending on whether it is a flowrate or an absolute amount. Therefore if three grams of catalyst were immobilised inside the reactor the entry would read “3:$$\texttt{g}$$:$$\texttt{a}$$”, while four moles per second of solvent being fed would read as “4:$$\texttt{m}$$:$$\texttt{f}$$”. Should the expression of the amount of catalyst only be possible in moles, then this format allows this to be easily accommodated by changing the flag to “:$$\texttt{m}$$” instead of “:$$\texttt{g}$$”. The amount of *group3* substances fed is also to be specified in scientific exponential notation.

The current version of RInChI allows for a species to appear in two places, say as reactant and as auxiliary, if a reactant for example also acts as solvent. This could lead to double-counting of masses when compiling the group1, 2 or 3 amount fields. Therefore, if a species appears more than once all entries but the first one for that species in the group1, group2 or group3 amount fields need to be marked appropriately. To this end, they are to be marked with an “x” followed by a colon and the group number and another colon and the index within that group corresponding to the first appearance of the species in the RInChI. Thus, the position where the amount fed can be found is indexed and links back to the first entry without registering the amount twice.

Furthermore, version 0.03 of the RInChI introduces empty fields instead of groups in the case of, for example, incomplete “half” reactions where no reactants or no products are listed. This can be observed in some cases in Reaxys. It is unclear if this is a faulty database entry or already the case in the paper. However, the standard provides for this to be generally applicable. If this is the case the field containing the amount fed of the corresponding group needs to be marked with a question mark as a place holder and left empty otherwise. Similarly, the number of amounts fed specified need to match the number of species specified in the respective group of the RInChI.

The volume of the reactor is to be expressed in terms of metres cubed, m^3^. In the case of a batch reaction it is to contain the expression “batch” instead. If it was a batch reaction the amounts of *group1* and *group2* substances given previously are absolute amounts, else they are flowrates. At the same time this provides valuable information about the scale of the reaction (bench, pilot or industrial).

Should the reaction have been carried out at several different sets of conditions (such as different temperatures) a separate *ProcAuxInfo* is to be published for each set and appended to the previous string.

Should no value be available for a given property the field in question still needs to be included in the string using the requisite separators, but the field itself is to contain a question mark (“?”) as space holder instead of a value.

The use of the *ProcAuxInfo* is demonstrated below for three published reactions carrying out palladium-catalyzed aziridination of aliphatic amines [40], a ruthenium oxide catalyzed oxidation of benzyl alcohol [41] and a Suzuki coupling. The first two have been chosen from the groups’ publications and the third has been randomly chosen from the reactions classified by Reaxys as Suzuki reactions [42]. However, only the data available in the published article or its published supplementary information were used in all three cases.

### Generation of *ProcAuxInfo*

#### Example 1

**Scheme 1:**
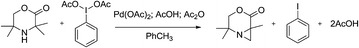
A case study of C–H activation reaction

The reaction is carried out between 3,3,5,5-tetramethylmorpholin-2-one (starting material) and (diacetoxyiodo)benzene as reactants forming 2,2,6-trimethyl-4-oxa-1-azabicyclo[4.1.0]heptan-5-one, iodobenzene and acetic acid. Toluene acts as solvent and palladium(II)acetate as catalyst and acetic acid and acetic anhydride as auxiliary substances as shown (Scheme 1).

Using the RInChI generator the following RInChI is generated for this reaction:
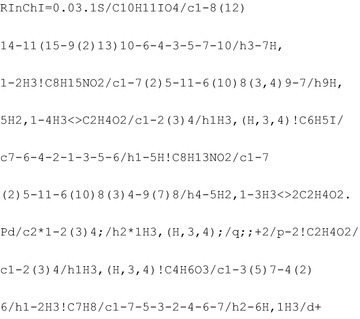


The reactor volume was 1 × 10^−5^ m^3^. All other required information on the reaction can be found in Tables 2, 3 and 4.

**Table 2:** The amounts of substances fed in the example 1

Compound	Amount fed (mol s^−1^)
3,3,5,5-Tetramethylmorpholin-2-one	8.3 × 10^−7^
(Diacetoxyiodo)benzene	8.3 × 10^−7^
Acetic acid	8.3 × 10^−6^
Palladium(II) acetate	4.2 × 10^−9^
Acetic anhydride	1.7 × 10^−6^
Toluene	1.5 × 10^−4^

**Table 3:** Conditions of reaction 1

Property	Value
Reaction temperature	393 K
Reaction pressure	6 × 10^6^ Pa
Yield	0.90

**Table 4:** Residence time: conversion pairs for the reaction 1

Residence time (s)	Conversion
60	0.06
120	0.14
180	0.20
240	0.32
300	0.40
360	0.52
420	0.70
480	0.90
540	1.00
600	1.00

The resulting *ProcAuxInfo* is thus given by:
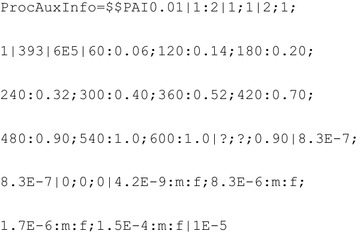


#### Example 2

**Scheme 2:**
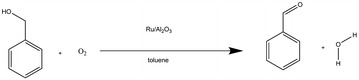
A case study of benzalcohol oxidation

This reaction oxidises benzyl alcohol into benzaldehyde and water with molecular oxygen as an oxidant, toluene as solvent and using ruthenium supported on aluminium oxide as a catalyst, as shown in Scheme 2.

InChIs are not currently able to represent ruthenium supported on aluminium oxide and thus considers them as separate species. This leads to the following RInChI:
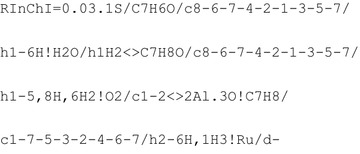


All required reaction data can be found in Tables 5, 6 and 7.

**Table 5:** Amounts of substances fed into the reaction 2

Compound	Amount fed
Benzyl alcohol	3.3 × 10^−5^ mol s^−1^
Toluene	3.1 × 10^−4^ mol s^−1^
Oxygen	4.9 × 10^−6^ mol s^−1^
Ruthenium	9 × 10^−3^ g
Aluminium oxide	0.991 g

**Table 6:** Conditions of the reaction 2

Property	Value
Reaction temperature	388 K
Reaction pressure	8 × 10^6^ Pa
Yield	0.25
Reactor volume	9 × 10^−4^ m^3^

**Table 7:** Residence time: conversion pairs for the reaction 2

Residence time (s)	Conversion
9	0.25

Allowance had to be made for the fact that the InChI standard is not able to represent ruthenium supported on aluminium oxide and thus required reporting of the two substances individually. This is a limitation in the InChI standard, which filters down to the RInChI and thus also impacts the ProcAuxInfo layer. Seeing as this limitation originates in the InChI it was not attempted to “fix” this limitation in the ProcAuxInfo layer as this would most likely be the wrong place for such an attempt.

The resulting *ProcAuxInfo* is thus given by:
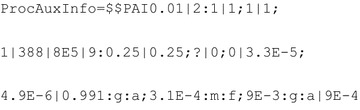


#### Example 3

**Scheme 3:**
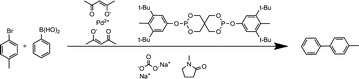
An example of a Suzuki–Miyaura reaction

For this example it was decided to encode a Suzuki–Miyaura reaction as this is a very common reaction in organic synthesis. A publication reporting the Suzuki–Miyaura reaction was chosen at random from Reaxys. The specific example [42] carries out a Suzuki–Miyaura reaction using phenylboronic acid and 4-bromotoluene as reagents to produce 4-phenyltoluene. It uses a phosphine ligand, *N*-methyl-2-pyrrolidinone as solvent and sodium carbonate as base as shown in Scheme 3.

Observing the reported reaction equation it is apparent that the equation is not balanced, since the byproduct species are missing. Another problematic factor is that the base is, at least partially, consumed during the reaction. Reporting it as an agent is, hence, not entirely accurate. Seeing as this example is translating the information provided in the paper this assumption is not questioned but the RInChI is generated taking account of the missing product species. Processing the information with the RInChI API yields the following RInChI:
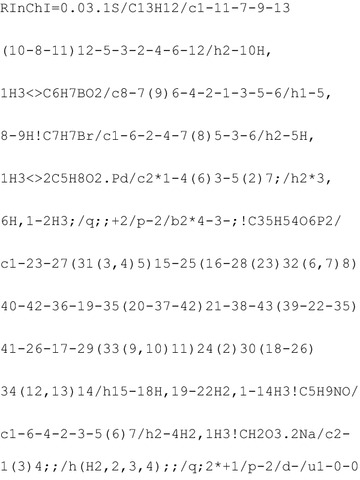


All required reaction data, as taken directly from the paper, can be found in Tables 8, 9 and 10.

**Table 8:** Amounts of substances fed into the reaction 3

Compound	Amount fed
Phenylboronic acid	1.1 × 10^−3^ mol
4-bromotoluene	1.0 × 10^−3^ mol
Phosphine ligand	2.2 × 10^−5^ mol
*N*-methyl-2-pyrrolidinone	3.1 × 10^−2^ mol
Palladium(II) acetylacetonate	2.2 × 10^−5^ mol
Sodium carbonate	Not reported

**Table 9:** Conditions of the reaction 3

Property	Value
Reaction temperature	363 K
Reaction pressure	Not reported
Yield	0.89
Reactor volume	Not reported

**Table 10:** Residence time: conversion pairs for the reaction 3

Residence time (s)	Conversion
.	.

From the way the data is reported we could deduce the limiting reactant and then the corresponding amounts of agents, which were reported as per cent. The amount of base was not reported at all. This highlights why a precisely defined set of information and the associated units are required when transmitting data, which would force the authors to complete the necessary data.

One might reasonably assume that the reaction was conducted at atmospheric pressure under reflux conditions as no pressure is given in the paper. However, doing so might run the risk of potentially establishing an erroneous assumption as fact. Hence, this is not done here and the pressure field is left blank. Similarly, a mention of the reactor volume is absent from the paper. The paper does not specify with regards to which species the yield is defined, but we could reasonably assume that this would be the limiting reactant.

The paper does not specify any side-reactions or by-products being formed so one might assume that all reacted reactant is converted into product, thus making conversion equal to yield. Given the fact that at least one product species is missing accepting this assumption at face value could be highly misleading. No reaction time is given; thus it is impossible to reliably deduce a residence time:conversion pair in Tables 7 and 10.

Taking the information thus extracted it is possible to produce a ProcAuxInfo string:
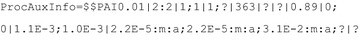


### Reverse lookup

#### Example 1

Looking up the InChIs contained in the RInChI using Chemspider [43] the following substances could be retrieved, see Table 11.

The species corresponding to “$\texttt{C8H13NO2/c1-7}(\texttt{2})\texttt{5-11-6}(\texttt{10})\texttt{8}(\texttt{3})\texttt{4-9}(\texttt{7})\texttt{8/h4-5H2},\texttt{1-3H3}$” could not be retrieved. Generating the InChI corresponding to 2,2,6-trimethyl-4-oxa-1-azabicyclo[4.1.0]heptan-5-one it was possible to verify that they were one and the same string. Thus, there seems to be a reverse translation issue in the InChI implementations, preventing accurate lookup of the InChI for this species.

Converting the *ProcAuxInfo* string the starting material is correctly recalled to be 3,3,5,5-tetramethyl-2-morpholinone and the stoichiometry is represented correctly. Temperature is correctly reported as 393 K and pressure as 6 bar. The residence time:conversion pairs match the supplied information and the yield is given as 90%. The amounts of species fed as well as the reactor volume also accurately match the provided information making the *ProcAuxInfo* recall successful.

#### Example 2

Again using the Chemspider [43] functionality to look up the InChIs the following substances were retrieved from the main RInChI, see Table 12.

All substances are recalled correctly, though it becomes apparent that InChI is not currently able to represent a catalyst supported on an inert support material requiring the two to be stored separately, leading to some loss of information.

The *ProcAuxInfo* string, correctly, identifies benzyl alcohol as the starting material and reports the reaction stoichiometry correctly. Temperature is reported as 388 K and pressure as 8 bar; both are correct. The residence time:conversion value and the yield are equally accurately represented. The *ProcAuxInfo* string accurately states that no *group1* materials were fed into the process and reports the feed rates of benzyl alcohol and oxygen as 3.3 × 10^−5^ mol s^−1^ and 4.9 × 10^−6^ mol s^−1^, respectively. The auxiliary amounts are also retrieved correctly, but, as previously alluded to, information is lost about the catalyst by forcing the reporting of Ru/Al_2_O_3_ as separate, isolated species. The reactor volume is stored as 9 × 10^−4^ m^3^, which is correct too (Tables 11 and 12).

**Table 11:** Reverse lookup results for the reaction 1 species

Group	Species
Reactants	(Diacetoxyiodo)benzene
	3,3,5,5-Tetramethyl-2-morpholinone
Products	Acetic acid
	Iodobenzene
	n/a
Auxiliaries	Palladium(II) acetate
	Acetic acid
	Acetic anhydride
	Toluene

**Table 12:** Reverse lookup results for the reaction 2 species

Group	Species
Products	Benzaldehyde
	Water
Reactants	Benzyl alcohol
	Molecular oxygen
Auxiliaries	Aluminum oxide
	Toluene
	Ruthenium

#### Example 3

Using the Chemspider functionality [43] it was possible to recall almost all species successfully as can be seen in Table 13. Two species are marked as “unknown”. This is, firstly, the second product which was already missing in the declaration of the RInChI and, secondly, the palladium ligand. This to a degree is also expected behaviour and the same problem was already observed in Example 1. Seeing as it is an unconventional molecule it is not surprising that it has not been reported with an associated InChI, causing the lookup to fail. Had the initial publication reported InChIs for the species contained therein, which would be the case if using the RInChI and ProcAuxInfo, this would not be an issue.

**Table 13:** Reverse lookup results for the reaction 3 species

Group	Species
Products	4-Phenyltoluene
	Unknown
Reactants	Phenylboronic acid
	4-Bromotoluene
Auxiliaries	Palladium acetylacetonate
	Unknown
	*N*-Methyl-2-pyrrolidone
	Sodium carbonate

Furthermore, it can be recalled from the ProcAuxInfo string that the reaction proceeded at 363 K and had a yield of 89% with respect to the starting material. No 4-phenyltoluene has been fed into the system; 1.1 × 10^−3^ mol of phenylboronic acid and 1.0 × 10^−3^ mol of 4-bromotoluene have been fed. Furthermore, 2.2 × 10^−5^ mol of phosphine ligand, 3.1 × 10^−2^ n-methyl-2-pyrrolidinone, 2.2 × 10^−5^ mol of palladium(II) acetylacetonate and an unspecified amount of sodium carbonate have been fed. This matches the data provided. No data is contained on the reactor volume.

This case study has also shown that the ProcAuxInfo is able to accurately store and recall data encoded in it. It does however also highlight that, like any data storage format, it is only able to store data that has been provided, and that in the case of many journal articles crucial data is simply either not being provided or provided in ways that are non-machine-readable. This illustrates why it is so important to establish a set of minimum data that need to be provided and a standard on how these are to be provided.

### Example application

As discussed in the introduction, it is possible to navigate the Network of Organic Chemistry. However, it is highly desirable to *evaluate* the identified routes according to some set of process metrics. Calculating this purely from the data currently contained in Reaxys would, at present, be impossible. If, however, the data contained in the proposed *ProcAuxInfo*, for example, is also taken into consideration, then the calculation is facile and it would be possible to automate many calculations. For example, calculation of E-factor (or other mass-based indicators) across reaction sequences built from Reaxys to allow their scoring, returning a greater amount of information to the user, would be possible, as shown in [9]. Other applications of the data contained in the *ProcAuxInfo*, such as analysis of deviation in operating conditions or flows of energy, are of course imaginable.

The E-factor was first proposed by Roger Sheldon in the 1980s and is a commonly used measure of a process’s efficiency, measuring the ratio of mass of waste produced to the mass of product produced [44]. In a paper from 2009 John Andraos presents an algorithm allowing the calculation of E-factors across a synthesis route [45]. This algorithm requires some modification to allow it to handle non 1:1 stoichiometry, yielding the following form [9]:

5 $$E_{total} = \frac{1}{{MR_{{p_{n} }} }}\mathop \sum \limits_{j} \left( {\frac{1}{{\mathop \prod \nolimits_{k}^{n \to j} \varepsilon_{k} }}\left( {\frac{{\nu_{{p_{j} }} }}{{\nu_{{mr_{j} }} }}\frac{{MR_{{p_{j} }} }}{{AE_{j} }}\times \left[ {SF_{j} - \frac{{\nu_{{mr_{j} }} }}{{\nu_{{p_{j} }} }}\varepsilon_{j} AE_{j} } \right] + \frac{{c_{j} + s_{j} + \omega_{j} }}{{n_{{mr_{j} }} }}} \right) } \right)$$

where *E* is the E-factor, *MR*_*p*_ the molecular weight of the desired product, *ɛ* is the yield with respect to the limiting reactant. The subscripts *j* and *n* relate to step number *j* in the synthesis route and the final step, respectively, where the sequence of steps is $\left( {1, \ldots ,j, \ldots ,n} \right)$. $\prod\nolimits_{k}^{n \to j} {\varepsilon_{k} }$ finally is the product of reaction yields along the reaction route from the current step to the final step ignoring any steps carried out prior to the current step; *c* is the mass of catalyst, *s* the mass of solvent, *ω* the mass of all other materials used in work-up and purification and $n_{{mr_{j} }}$ the experimental mole scale of the limiting reagents in step *j*; *ν*_*p*_ and *ν*_*mr*_ are the stoichiometric coefficients of desired product and limiting reactant, respectively; *AE* is the atom economy taking reaction stoichiometry into account and $SF = 1 + \frac{excess\,mass\,of\,reagents}{stoichiometric\,mass\,of\,reagents}$ is the stoichiometric factor [9].

The only additional piece of information required is the molecular masses of the involved species, which are routinely available from Reaxys. Thus, applying Eq. (5) to Example 1 yields an E-factor of 127.4. E-factors for commodity products range between 5 and 50 while those in the pharmaceutical industry can exceed 100 [46]. Therefore, the obtained E-factor is not widely wrong, being in the expected range.

## Conclusions

A large gap currently exists in the chemical data reporting standards rendering much of the currently available data unusable by algorithmic analyses. This paper has proposed and demonstrated an extension to the currently existing RInChI, which itself is an extension of the InChI. The proposed extension, termed *ProcAuxInfo*, is able to store additional pieces of process information on operating conditions, material flows and experimental setup, which allow a deeper mining of the data currently available in journals and databases. This work has been able to recall all data stored in the *ProcAuxInfo* correctly, though has in two instances suffered from slight data loss due to issues of the underlying current InChI implementation. Nonetheless, it is a useful extension to the RInChI as it allows the explicit storage of process information. If adopted and routinely requested by the publishing industry at the point of manuscript acceptance, it would ensure that abstraction of data from journals becomes more accurate and that more of the data contained in publications remains available to the community.
